# Periodontal Tissue Regeneration Using Fibroblast Growth Factor -2: Randomized Controlled Phase II Clinical Trial

**DOI:** 10.1371/journal.pone.0002611

**Published:** 2008-07-02

**Authors:** Masahiro Kitamura, Keisuke Nakashima, Yusuke Kowashi, Takeo Fujii, Hidetoshi Shimauchi, Takashi Sasano, Toshi Furuuchi, Mitsuo Fukuda, Toshihide Noguchi, Toshiaki Shibutani, Yukio Iwayama, Shogo Takashiba, Hidemi Kurihara, Masami Ninomiya, Jun-ichi Kido, Toshihiko Nagata, Takafumi Hamachi, Katsumasa Maeda, Yoshitaka Hara, Yuichi Izumi, Takao Hirofuji, Enyu Imai, Masatoshi Omae, Mitsuru Watanuki, Shinya Murakami

**Affiliations:** 1 Osaka University Dental Hospital, Suita, Japan; 2 Dental Hospital, Health Sciences University of Hokkaido, Ishikari-Tobetsu, Japan; 3 Medical and Dental Clinic, Health Sciences University of Hokkaido, Sapporo, Japan; 4 Tohoku University Dental Hospital, Sendai, Japan; 5 Aichigakuin University Dental Hospital, Nagoya, Japan; 6 Asahi University Dental Hospital, Mizuho, Japan; 7 Okayama University Hospital of Dentistry, Okayama, Japan; 8 Hiroshima University Hospital of Dentistry, Hiroshima, Japan; 9 Tokushima University Dental Hospital, Tokushima, Japan; 10 Kyushu University Dental Hospital, Fukuoka, Japan; 11 Nagasaki University Hospital, Attached School of Dentistry, Nagasaki, Japan; 12 Kagoshima University Dental Hospital, Kagoshima, Japan; 13 Fukuoka Dental College Hospital, Fukuoka, Japan; 14 Osaka University Hospital, Suita, Japan; 15 Izumisano Municipal Hospital, Rinku General Medical Center, Izumisano, Japan; 16 Kaken Pharmaceutical Co., Ltd., Tokyo, Japan; University of Michigan, United States of America

## Abstract

**Background:**

The options for medical use of signaling molecules as stimulators of tissue regeneration are currently limited. Preclinical evidence suggests that fibroblast growth factor (FGF)-2 can promote periodontal regeneration. This study aimed to clarify the activity of FGF-2 in stimulating regeneration of periodontal tissue lost by periodontitis and to evaluate the safety of such stimulation.

**Methodology/Principal Findings:**

We used recombinant human FGF-2 with 3% hydroxypropylcellulose (HPC) as vehicle and conducted a randomized double-blinded controlled trial involving 13 facilities. Subjects comprised 74 patients displaying a 2- or 3-walled vertical bone defect as measured ≥3 mm apical to the bone crest. Patients were randomly assigned to 4 groups: Group P, given HPC with no FGF-2; Group L, given HPC containing 0.03% FGF-2; Group M, given HPC containing 0.1% FGF-2; and Group H, given HPC containing 0.3% FGF-2. Each patient underwent flap operation during which we administered 200 µL of the appropriate investigational drug to the bone defect. Before and for 36 weeks following administration, patients underwent periodontal tissue inspections and standardized radiography of the region under investigation. As a result, a significant difference (p = 0.021) in rate of increase in alveolar bone height was identified between Group P (23.92%) and Group H (58.62%) at 36 weeks. The linear increase in alveolar bone height at 36 weeks in Group P and H was 0.95 mm and 1.85 mm, respectively (p = 0.132). No serious adverse events attributable to the investigational drug were identified.

**Conclusions:**

Although no statistically significant differences were noted for gains in clinical attachment level and alveolar bone gain for FGF-2 groups versus Group P, the significant difference in rate of increase in alveolar bone height (p = 0.021) between Groups P and H at 36 weeks suggests that some efficacy could be expected from FGF-2 in stimulating regeneration of periodontal tissue in patients with periodontitis.

**Trial Registration:**

ClinicalTrials.gov NCT00514657

## Introduction

Periodontitis, evoked by the bacterial biofilm (dental plaque) that forms around teeth, progressively destroys the periodontal tissue supporting the teeth, including the periodontal ligament, cementum, alveolar bone and gingiva. Ultimately, this chronic inflammatory disease can lead to loss of the affected teeth [1]–[3]. All over the world, this disease remains highly prevalent [4] and is considered to threaten quality of life (QOL) for middle-aged and older populations as far as “oral” functions are concerned. Some success has been achieved in suppressing progression of periodontitis by mechanically removing bacterial biofilm, the very cause of the disease. However, removal of the cause, bacterial plaque, with conventional periodontal and/or surgical treatments can, at best, reduce pocket depth and diminish inflammation in the affected region. No such treatment can ever regenerate lost periodontal tissue or normal structure and functionality. Considering that the “mouth” and “teeth” have various aesthetic and functional roles to play, establishing a brand-new treatment that enables the regeneration and rebuilding of periodontal tissue once destroyed by periodontal disease represents a task of tremendous importance.

To regenerate periodontal tissue destroyed by periodontitis, the chain of events requires stimulation of cementoblasts and osteoblasts into differentiation on the dental root and alveolar bone surfaces facing the region of periodontal tissue defect, followed by regeneration of the cementum and alveolar bone. Collagen fascicles produced by the periodontal ligament fibroblasts should then be embedded into those regenerated hard tissues, to rebuild new tissue to support teeth. Researchers have recently confirmed the existence of mesenchymal stem cells within the periodontal ligament, one of the cornerstones of periodontal tissue. These stem cells can differentiate into cells such as cementoblasts and osteoblasts [5]. Using the biological potentials of those stem cells to stimulate the regeneration of periodontal tissue is now being recognized as clinically possible. Some researchers are already trying to establish new treatments to accelerate the regeneration of periodontal tissue by local application of human recombinant cytokines to stimulate proliferation and differentiation into hard-tissue forming cells of undifferentiated mesenchymal cells among periodontal ligament cells. Direct local application of a combination of factors such as platelet-derived growth factor (PDGF) and insulin-like growth factor (IGF)-I [6], bone morphogenetic protein (BMP)-2 [7], [8], transforming growth factor (TGF)-β [9], osteogenic protein (OP)-1 [10] and brain-derived neurotrophic factor (BDNF) [11] to artificial defects in periodontal tissue made in laboratory animals reportedly stimulates and promotes regeneration of regional periodontal tissue. In addition, the efficacy of PDGF-BB plus β-tricalcium phosphate (β-TCP, an osteoconductive scaffold) for periodontal tissue regeneration in human has recently been reported [12].

Fibroblast growth factor (FGF)-2 displays potent angiogenic activity and mitogenic ability on mesenchymal cells. To date, FGF-2 has been reported as efficacious in regenerating periodontal tissue in models of artificial defect of periodontal tissue in beagles and non-human primates (*Macaca fascicularis*) and in a model of surgically created periodontitis in beagles [13]–[15].

The present clinical trial used hydroxypropylcellulose (HPC)-based FGF-2 as the investigational agent. The purpose of this trial was to both clarify the activity of FGF-2 to regenerate periodontal tissue in periodontitis patients and to confirm drug safety. This study was a randomized, double-blinded clinical trial (Phase II) involving placebos and multiple dental facilities in compliance with good clinical practice (GCP) guidelines, representing the first trial to examine the efficacy and safety of FGF-2 in periodontitis patients with concurrent control of dose-response relationships.

The periodontium that supports teeth displays a tissue structure wherein the alveolar bone (hard tissue surrounding dental roots) is covered by the gingiva (soft tissue), and “true regeneration” thus involves the regeneration of both hard and soft tissues. To improve tooth support, regenerating hard tissues including alveolar bone is crucial. Hence, in the present study, under the assumption that FGF-2 would regenerate both hard and soft tissues, the rate of increase in alveolar bone height was established as the most important outcome measure. Furthermore, to confirm soft-tissue regeneration, the millimeter of clinical attachment level (CAL) regained was added as a main outcome measure.

Including recruitment of subjects, the clinical trial was performed from December 1, 2001 to September 29, 2004.

## Methods

The Protocol for this trial and supporting CONSORT checklist are available as supporting informations; see Checklist S1 and Protocol S1.

This was a randomized, double-blinded, clinical trial of dose responses including placebo comparison, involving 13 dental facilities. Study protocols were approved prior to initiation of the study by the institutional review boards of the respective participating facilities.

### 1. Participants

Patients with periodontitis visiting any of the 13 dental institutions listed in Table 1 were requested to participate. In compliance with GCP guidelines, prospective 91 patients who provided written informed consent underwent clinical inspection and oral cavity diagnosis. Among 91 patients 80 patients who satisfied the selection and exclusion criteria described in Tables 2 and 3 were finally registered. Each subject received a standard initial preparation, including oral hygiene instruction, full-mouth scaling and root planing before surgical treatment, to minimize bacterial insult and reduce variability between lesions at baseline. Using oral radiographs and periodontal tissue inspection results, regions of investigation were determined as 2- or 3-walled vertical periodontal tissue defects ≥3 mm apical to the remaining alveolar bone crest.

**Table 1:** The 13 trial dental facilities and the investigators

Trial facilities	Investigators	Number of patients
Dental Hospital, Health Sciences University of Hokkaido	Yusuke Kowashi	4
Medical and Dental Clinic, Health Sciences University of Hokkaido	Takeo Fujii	9
Tohoku University Dental Hospital	Hidetoshi Shimauchi	6
Aichigakuin University Dental Hospital	Mitsuo Fukuda	7
Asahi University Dental Hospital	Toshiaki Shibutani	6
Osaka University Dental Hospital	Masahiro Kitamura	6
Okayama University Hospital of Dentistry	Shogo Takashiba	11
Hiroshima University Hospital of Dentistry	Hidemi Kurihara	3
Tokushima University Dental Hospital	Jun-ichi Kido	12
Kyushu University Dental Hospital	Takafumi Hamachi	2
Nagasaki University Hospital Attached School of Dentistry	Yoshitaka Hara	6
Kagoshima University Dental Hospital	Yuichi Izumi	8
Fukuoka Dental College Hospital	Takao Hirofuji	0
		80

**Table 2:** Criteria for selecting subjects

1)	Those diagnosed as having, from radiography and other results, 2- or 3-walled vertical intrabony defect as being measured at ≥3 mm apical to the remaining alveolar bone crest
2)	Those who have accomplished initial preparation and have been showing good compliance
3)	Those with mobility of the tooth to investigate of Degree 2 or less and with width of attached gingiva for which the existing Guided Tissue Regeneration (GTR) treatment is considered appropriate (Those with no width of keratinized gingival is not eligible)
4)	Those for whom supportive periodontal treatment (SPT) is applicable, in accordance with usual post-operative procedures following flap operation and GTR treatment
5)	Those whose oral hygiene is well established and who are able to perform appropriate tooth brushing following instructions of the investigators and/or sub-investigators after investigational drug administration
6)	Those ≥20-years-old and <65-years-old
7)	Those who understand the purposes of the trial and are capable of making an independent decision to comply with trial requirements
8)	Those who are able to visit their hospitals in accordance with the trial schedule

We selected those patients who met the criteria listed above, from those who the investigators and/or sub-investigators determined were in need of flap operation.

**Table 3:** Criteria for excluding subjects

1)	Those administered a calcium antagonist during the 4 weeks preceding administration of the investigational drug
2)	Those in need of administration of adrenal cortical steroid (equivalent to>20 mg/day of Predonin) within 4 weeks after investigational drug administration
3)	Those scheduled to undergo a surgical operation in the vicinity of the tooth to investigate within 36 weeks after investigational drug administration
4)	Those with coexisting mental or consciousness disorder
5)	Those with coexisting malignant tumour or history of the same
6)	Those with coexisting diabetes (HbA_1C_ >6.5%)
7)	Those in an extremely poor nutritional condition (serum albumin concentration <2 g/dL)
8)	Those with ≥200 mL of blood drawn during the 4 weeks preceding investigational drug administration
9)	Those administered another investigational drug during the 24 h preceding investigational drug administration
10)	Those with coexisting disorder of the kidney, liver, blood and/or circulatory system (Grade 2 or above)
11)	Those who are either pregnant, possibly pregnant or breast-feeding, or who hope to become pregnant during the period of the trial
12)	Those with a previous history of hypersensitivity to a protein drug
13)	Others who the investigators or sub-investigators determined as unsuitable for the trial

### 2. Interventions, Design and Procedure

This trial employed recombinant human FGF-2 (Code No. KCB-1; Kaken Pharmaceutical Co., Ltd., Tokyo, Japan) produced by genetic recombination that introduced the gene for human FGF-2 into *Escherichia coli*. To improve the operability of drug administration to the region of alveolar bone defect, before administration we mixed freeze-dried FGF-2 with 3% HPC, a colorless and viscid solution, and prepared the gel-like investigational drug for this clinical trial (Code No. KCB-1D). FGF-2 concentration in the investigational drug was then prepared to 0% (placebo), 0.03%, 0.1% or 0.3% and administered to the region of investigation within 2 h of preparation. Before the start and after completion of investigational drug administration, a third-party organization (University of Shizuoka, Shizuoka, Japan) measured FGF-2 concentrations for each group to ascertain that FGF-2 concentrations in vials were accurate according to good manufacturing practice standards.

**Figure 1:**
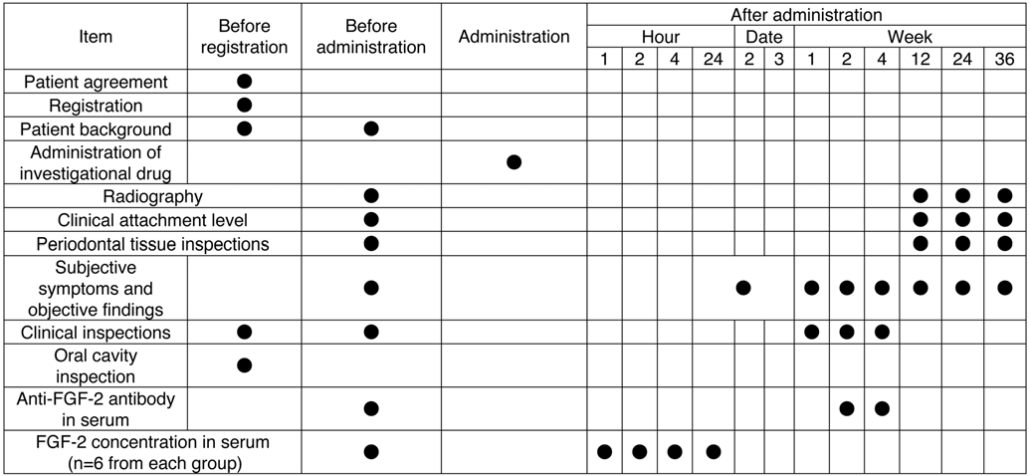
Schedule of the clinical trial. We randomly allocated the 80 patients into 4 groups (n = 20 each): 1) a placebo group (Group P); 2) a group administered 0.03% FGF-2 (Group L); 3) a group administered 0.1% FGF-2 (Group M); and 4) a group administered 0.3% of FGF-2 (Group H). The clinical trial was then conducted in accordance with the clinical trial schedule. We also measured FGF-2 concentrations in the blood serum of 6 patients randomly chosen from each of the 4 groups, before and then 1 h, 2 h and 4 h after administration of the investigational drug.

The clinical trial was conducted according to the schedule shown in Figure 1. The 80 patients were registered at the Registration Center (Adjust Co., Ltd., Sapporo, Japan) and then randomly assigned to the following 4 groups: Group P, placebo group administered HPC containing no FGF-2; Group L, administered HPC containing 0.03% FGF-2; Group M, administered HPC containing 0.1% FGF-2; and Group H, administered HPC containing 0.3% FGF-2.

All flap operations were performed in accordance with the modified Widman procedure. The proposed surgical area was anesthetized using local anesthetic. Following intracrevicular incision, buccal and lingual full-thickness (mucoperiosteal) flaps were elevated. Following reflection of the mucoperiosteal flap, all granulation tissue associated with the bone defect was removed. Subgingival soft and hard deposits on the root surface were removed utilizing both hand and ultrasonic instrumentation to ensure thorough degranulation and root planing. After that, 200 µL of investigational drug was administered to the bone defect region described above. No specific root conditioning was performed.

Next, at 1, 2 and 4 weeks after administration, the same clinical inspections were performed as before administration, and anti-FGF-2 antibodies in serum 2 and 4 weeks after administration were measured. At 12, 24 and 36 weeks following administration, standardized radiographs were taken, periodontal tissues were inspected and subjective symptoms and objective findings were observed. In addition, 6 patients from each of the groups were randomly selected and blood samples were drawn. At 1, 2 and 4 h after administering the investigational drug, FGF-2 concentrations in serum were measured.

### 3. Randomization

An independent organization, the Registration Center (Adjust Co., Ltd., Sapporo, Japan), was used to keep treatment allocation inaccessible to any patients or other individuals involved in the trial. The Registration Center created an allocation table in which a block size of 4 cases per block was allocated to investigational drugs comprising placebo (Group P), 0.03% FGF-2 (Group L), 0.1% FGF-2 (Group M) or 0.3% FGF-2 (Group H). According to this allocation table, a label indicating the corresponding drug number was attached to each and every vial of drug. After drugs were allocated, the Registration Center sealed and kept the allocation table in confidence until the clinical trial was completed. Freeze-dried drugs for Groups P, L, M and H were indistinguishable based on appearance.

Investigators at each facility checked all inclusion and exclusion criteria and registered patients one at a time by faxing patient information obtained under informed consent to the Registration Center. The center again checked the documents to make sure that each subject had satisfied all inclusion and exclusion criteria, then randomly allocated subjects as necessary to receive drugs based on a single block consisting of one drug sample each from Groups P, L, M, and H. The assigned drug numbers were then faxed back to the investigators. The blind was not broken until this clinical trial was completely finished.

### 4. Outcome measures

Main outcome measures prespecified in the study protocol comprised: rate of increase in alveolar bone height; and millimeter of CAL regained. In addition, we examined whether and to what extent adverse events emerged for which causal relationships with the investigational drug were not ruled out before breaking the blind. We set rate of increase in alveolar bone height as the most statistically important outcome (primary outcome). Probing depth (PD), bleeding on probing (BOP), gingival index (GI), tooth mobility (MO), gingival recession (REC), plaque index (PlI), and width of keratinized gingiva (KG) were selected as secondary outcome measures.

1) Standardized radiography for regions of investigation

Our geometrically standardized radiography employed dental film (Kodak InSight Super Poly-Soft; Eastman Kodak Company, New York, USA) and photograph indicators (Cone Indicator-II; Hanshin Technical Laboratory, Nishinomiya, Japan) customized with resin stents.

**Figure 2:**
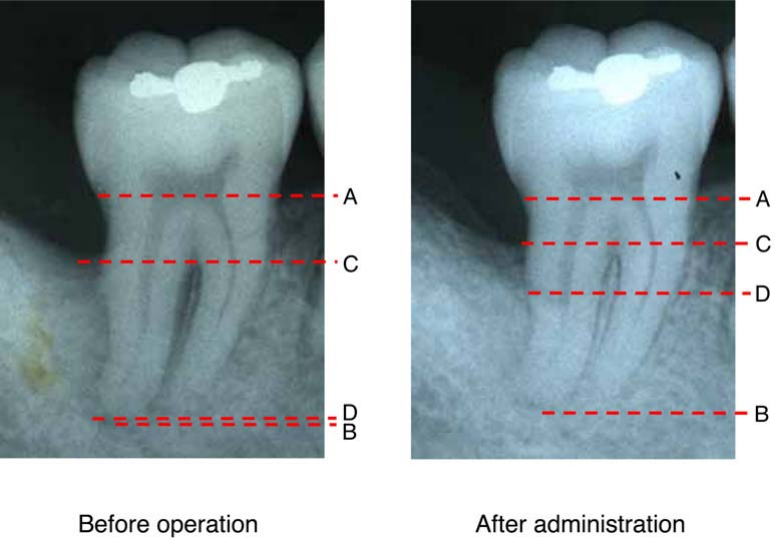
Measured points of alveolar bone height using standardized radiographs. Standardized dental radiographs taken before and after FGF-2 administration in one subject (a 29-year-old man) given 0.3% FGF-2. Points A, B, C and D represent the cementoenamel junction, apex, remaining alveolar bone crest and bottom of the bone defect, respectively. The examiners measured tooth axis heights between Points A and B, Points A and C, and Points A and D on the X-ray for each patient. To adjust for slight errors due to imaging, measurements for 5 examiners were multiplied by A-B ratio of before to after administration to correct A-B, A-C and A-D after administration (adjusted A-B, A-C and A-D, respectively). Rate of increase in alveolar bone height was derived from the following calculation formula. [(A-D before administration) - (adjusted A-D after administration)] by C-D before administration. On this radiography, C-D before administration, A-D before administration and, adjusted A-D after administration measured 9.00 mm, 12.80 mm and 5.93 mm, respectively. These values assigned to the above formula, we obtained the rate of increase in alveolar bone height as follows. The rate of increase in alveolar bone height (%) = 100(12.80–5.93)/9.00 = 76.35.

Five doctors specializing in dental radiology from the Department of Oral Diagnosis at Tohoku University Graduate School of Dentistry independently measured rate of increase in alveolar bone height using the methods described in Figure 2. Errors caused by slight variation in angulations of X-ray imaging were corrected based on the distance between two immobile anatomical reference points. The median of 5 measurements taken from the same image was then selected for efficacy analysis. Before making measurements in the present study, X-ray images were read to measure intra- and interexaminer variations. Each of the 5 doctors measured the same sample 5 times to calculate coefficients of variation. The results showed that intra- and interexaminer coefficients of variation were both 3%, confirming the absence of marked variations.

2) Inspection of periodontal tissue around the tooth under investigation

We measured the items shown in Table 4 at 6 positions (mesiobuccal, buccal, distobuccal, mesiolingual, lingual and distolingual) around each tooth under investigation.

**Table 4:** Periodontal tissue inspection

1)	Clinical attachment level (CAL): A stent was prepared for each subject. Using as the control point the cementoenamel junction or margin of the restorative material, distance between the control point and bottom of the gingival sulcus was measured for each test subject, using the same periodontal probe.
2)	Probing depth (PD): Simultaneously with CAL measurement, we measured distance from the gingival margin to the bottom of the gingival sulcus for each subject using the same periodontal probe.
3)	Bleeding on probing (BOP; + or −): The presence of bleeding was checked 10 s after probing.
4)	Gingival index (GI): GI was determined as described by Löe and Silness.^16^
5)	Mobility of tooth (MO): MO was determined as described by Miller.^17^
6)	Recession of gingiva (REC): Using as the control point the cementoenamel junction or margin of the restorative material, distance between the control point and gingival margin was measured for each subject, using the same periodontal probe.
7)	Plaque index (PlI): PlI was determined as described by Silness and Löe.^18^
8)	Width of keratinized gingiva (KG): The shortest distance between the coronal gingival margin and mucogingival junction was measured for each subject, using the same periodontal probe.

All examiners used PCP-UNC-15 periodontal probes (Hu-Friedy, Chicago, IL). We held meetings with each investigator from all of the participating facilities. In addition, a start-up meeting in which all investigators from a single facility participated was held at each facility. In these meetings, the protocol for this clinical trial was confirmed and clinical evaluations were standardized between facilities. In all facilities, the same person (MW) explained the detailed methods of probing inspections to all investigators and confirmed reproducibility and consistency for each investigator. Furthermore, prior to initiating baseline measurements, intra- and interexaminer calibrations were performed on patients at each facility to ensure reproducibility and consistency by each investigator. Each patient was examined by the same examiner at every recall visit throughout this clinical trial.

### 5. Safety evaluation

1) Observation of subjective symptoms and objective findings

Medical findings for both the oral cavity and whole body were confirmed by interview and visual inspection.

2) Clinical inspections

A clinical testing company (SRL Medisearch Inc., Tokyo, Japan) measured the inspection items (see Table S1 of supporting items). In cases where we discovered unusual changes in any of the clinical inspection values listed within 4 weeks after administration of the investigational drug, a follow-up survey was conducted.

3) Measurement of anti-FGF-2 antibody levels in serum

The Pharmacokinetics Department of Kaken Pharmaceutical Co., Ltd. measured levels of anti-FGF-2 antibody (IgG) in serum using ELISA.

4) Measurement of FGF-2 concentration within serum

The Metabolism Research Department of Kaken Pharmaceutical Co., Ltd. measured FGF-2 concentrations in serum using ELISA.

### 6. Sample size calculation

The effect of a combination drug comprising recombinant human PDGF-BB and IGF-I in humans on periodontal regeneration has already been reported [19]. In PDGF-BB/IGF-I-treated subjects (n = 16), mean (±standard error of mean) bone fill was 18.5±7% for control sites (surgery alone) and 42.3±9% for PDGF-BB/IGF-I sites with a mean difference of 23.8%. Assuming a rate of increase for placebo control of 20% (standard deviation, 28%) in alveolar bone of the defect region, the planned sample size of 20 patients in each group would provide 90% power to detect any clinically relevant treatment difference of 30% at a two-tailed significance level of 0.05.

### 7. Statistical methods

For analysis, we employed SAS version 8.2 software (SAS Institute Inc., Carey, North Carolina, USA). The level of statistical significance was set at p<0.05 in advance. Data analysis covered those patients administered the randomly allocated investigational drugs. In analyses concerning efficacy, those patients found to have either 1- or 4-walled intrabony defect during surgery after allocation were excluded. To statistically compare the 3 dose groups in terms of rate of increase in alveolar bone mass with the placebo group, the Dunnett option was used based on the Mixed procedure in the SAS system, in which adjusted p-values were computed for multiple comparisons, and analysis for rates of increase during follow-up was performed using repeated-measures analysis of variance with the Mixed procedure.

## Results

### 1. Patient characteristics at the beginning of the trial (baseline characteristics)

**Figure 3:**
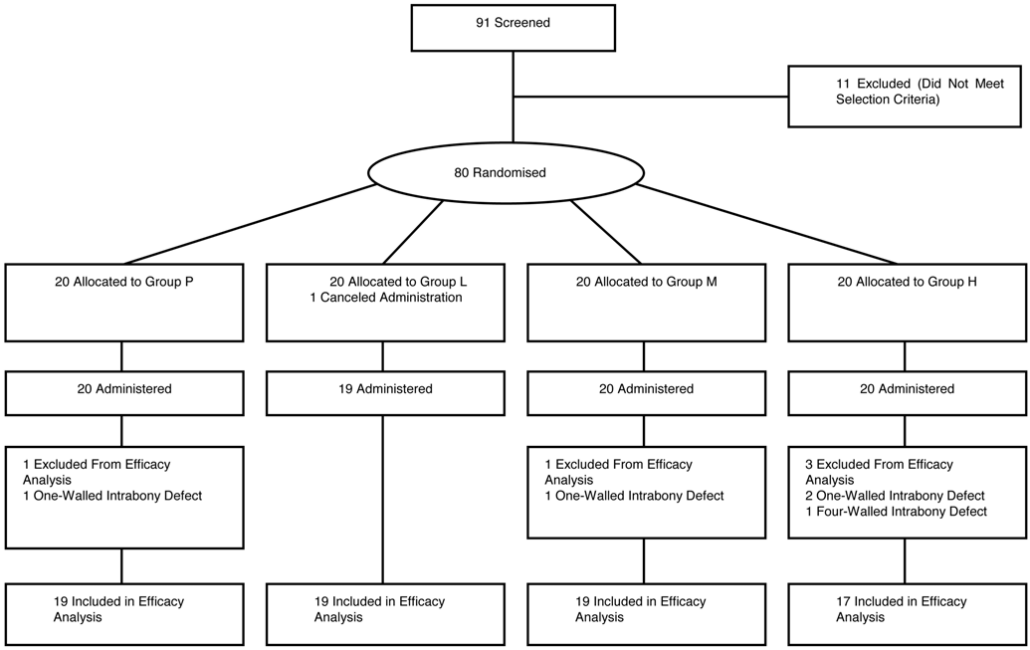
Flow of patients through the study.

Figure 3 shows flow of patients through the study. The 91 patients screened as subjects were consenting periodontitis patients for whom periodontal tissue regeneration therapy was indicated by investigators based on these criteria among a large number of potential subjects. Following the exclusion of 11 of these 91 patients, a final total of 80 patients were enrolled as subjects in the present clinical trial. The 11 patients were excluded due to findings on clinical inspection that could not have been determined by investigators during clinical periodontal diagnosis, or due to withdrawal of consent to participate. The 80 patients were then randomly assigned to 4 groups of 20 patients each. Table 5 shows the baseline characteristics of patients.

**Table 5:** Patient characteristics

Item	Classification	Group P	Group L	Group M	Group H
Numbers of patients		20	19	20	20
Sex (% of patients)	Male	55.0	36.8	25.0	35.0
	Female	45.0	63.2	75.0	65.0
Age (years)	Mean (SD)	49.2 (8.9)	46.2 (11.1)	46.8 (10.3)	47.7 (10.5)
Coexisting disease (% of patients)	No	70.0	57.9	75.0	85.0
	Yes	30.0	42.1	25.0	15.0
Previous history	No	75.0	73.7	60.0	60.0
	Yes	25.0	26.3	40.0	40.0
Smoking habit	No	75.0	89.5	80.0	70.0
	Yes	25.0	10.5	20.0	30.0
Region of administration (Major classification) (% of patients)	Maxilla	40.0	57.9	55.0	60.0
	Mandible	60.0	42.1	45.0	40.0
Region of administration (Minor classification) (% of patients)	Anterior tooth	25.0	21.1	25.0	30.0
	Premolar	35.0	42.1	40.0	40.0
	Molar	40.0	36.8	35.0	30.0
Depth of bone defect at operation (mm)	Mean (SD)	4.7 (1.5)	4.8 (2.4)	4.6 (1.7)	5.7 (2.6)
Classification of bone defect (% of patients)	1-walled	5.0	0.0	5.0	10.0
	2-walled	50.0	47.4	70.0	50.0
	3-walled	40.0	47.4	25.0	30.0
	4-walled	0.0	0.0	0.0	5.0
	2/3-walled	0.0	5.3	0.0	5.0
	1/2-walled	5.0	0.0	0.0	0.0
Treatment to tooth of investigation (% of patients)	No	60.0	57.9	55.0	55.0
	Yes	40.0	42.1	45.0	45.0
Existent of dental pulp (% of patients)	No	15.0	15.8	20.0	25.0
	Yes	85.0	84.2	80.0	75.0
Clinical attachment level (mm)	Mean (SD)	9.3 (2.2)	8.4 (2.7)	8.4 (2.8)	8.3 (3.0)
Probing depth (mm)	Mean (SD)	5.7 (1.2)	5.4 (1.6)	5.1 (2.0)	5.8 (1.7)
Recession (mm)	Mean (SD)	2.4 (1.8)	2.1 (1.5)	2.2 (2.3)	1.7 (1.5)
Width of keratinized gingival (mm)	Mean (SD)	4.9 (2.1)	4.3 (1.9)	4.5 (2.2)	5.3 (2.7)
Gingival bleeding index (% of patients)	−	10.0	15.8	20.0	5.0
	+	90.0	84.2	80.0	95.0
Gingival index (% of patients)	0	35.0	21.1	25.0	10.0
	1	30.0	47.4	40.0	45.0
	2	35.0	31.6	35.0	45.0
Mobility of tooth (% of patients)	0	65.0	57.9	50.0	40.0
	1	35.0	36.8	50.0	55.0
	2	0.0	5.3	0.0	5.0
Plaque index (% of patients)	0	50.0	42.1	80.0	60.0
	1	35.0	57.9	20.0	30.0
	2	15.0	0.0	0.0	10.0

### 2. Evaluation of efficacy

**Figure 4:**
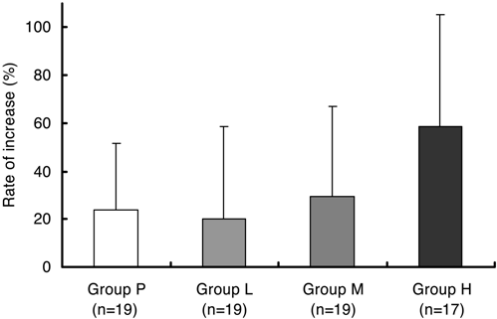
Rates of increase in alveolar bone height in cases of 2- and 3-walled intrabony defects. We compared rates of increase in alveolar bone height at 36 weeks after FGF-2 administration among Group P (19 placebo cases), Group L (19 cases administered 0.03% FGF-2), Group M (19 cases administered 0.1% FGF-2) and Group H (17 cases administered 0.3% FGF-2). This figure shows mean increase rates (%) and standard deviations of alveolar bone height. While no significant difference was observed between Groups L and M and P, Group H showed significantly increased (p = 0.021) alveolar bone height in the bone defect region compared to Group P.

Rate of increase in alveolar bone height at 12, 24 and 36 weeks after FGF-2 administration are shown in Table 6. A significant difference (p = 0.021) was only identified between Group P and Group H at 36 weeks. The detailed data at 36 weeks are shown in Fig. 4 and Table 7. Adjusted mean differences from Group P were also calculated as least square mean (LSMean) differences based on two-way analysis of variance or analysis of covariance (data not shown). Adjusted mean differences for gender, site of investigational drug administration (maxilla or mandible), CAL, REC, GI, MO, PlI and type of bone defect mostly resembled raw mean differences and the lower 95% confidence limits of LSMean difference (Group P vs. H) was above zero (0<lower 95% confidence limit). These results indicate that baseline characteristic imbalances between groups had no influence on evaluation of efficacy. Regarding the CAL regained (Table 8), REC, KG, MO and PlI (see Table S2 of supporting items), no significant differences existed between the 4 groups. Although PD, GI and BOP prevalence all decreased with time following periodontal surgical treatment in the 4 groups (see Table S3 of supporting items), no significant differences were noted between these groups.

**Table 6:** Changes with time in alveolar bone height

		Group P (n = 19)	Group L (n = 19)	Group M (n = 19)	Group H (n = 17)
rate of increase (%)	12 weeks	6.90 (20.12)	2.03 (18.79)	−0.82 (33.1)	13.86 (33.03)
	24 weeks	17.44 (28.48)	12.33 (27.50)	12.59 (23.67)	35.58 (40.35)
	36 weeks	23.92 (27.52)	20.19 (38.09)	29.39 (37.71)	*58.62 (46.74)
millimeter increase	12 weeks	0.28 (0.80)	0.07 (0.58)	0.15 (0.71)	0.55 (1.37)
	24 weeks	0.67 (1.25)	0.38 (0.97)	0.53 (0.71)	1.21 (1.57)
	36 weeks	0.95 (1.26)	0.54 (1.26)	1.06 (1.16)	1.85 (1.75)

Mean and standard deviations are shown. ^*^A significant difference (p = 0.021) was only identified between Group P and Group H at 36 weeks in rate of increase in alveolar bone height.

**Table 7:** Changes in alveolar bone height at 36 weeks

		Group P (n = 19)	Group L (n = 19)	Group M (n = 19)	Group H (n = 17)
rate of increase (%)	Mean (SD)	23.92 (27.52)	20.19 (38.09)	29.39 (37.71)	58.62 (46.74)
	Mean differences		−3.73	5.47	34.7
	from Group P (95%CI)		(−28.22–20.77)	(−19.02–29.97)	(9.50–59.91)
	Adjusted p value*		0.981	0.945	0.021
millimeter increase	Mean (SD)	0.95 (1.26)	0.54 (1.26)	1.06 (1.16)	1.85 (1.75)
	Mean differences		−0.41	0.11	0.90
	from Group P (95%CI)		(−1.24–0.42)	(−0.69–0.91)	(−0.13–1.92)
	Adjusted p value*		0.678	0.990	0.132

* Adjusted for multiple comparisons based on Dunnett's test.

**Table 8:** Clinical Attachment Level (CAL) regained at 36 weeks

		Group P (n = 19)	Group L (n = 19)	Group M (n = 19)	Group H (n = 17)
mm of CAL regained	Mean (SD)	2.63 (1.54)	2.00 (2.08)	2.02 (2.08)	2.18 (1.33)
	Mean differences		−0.63	−0.61	−0.46
	From Group P (95%CI)		(−1.84–0.57)	(−1.81–0.60)	(−1.43–0.53)
	Adjusted p value*		0.573	0.604	0.792
% of CAL regained	Mean (SD)	29.65 (17.00)	24.03 (25.31)	24.20 (28.27)	29.69 (23.14)
	Mean differences		−5.62	−5.45	0.04
	from Group P (95%CI)		(−19.81–8.60)	(−20.79–9.90)	(−13.61–13.70)
	Adjusted p value*		0.810	0.823	1.000

* Adjusted for multiple comparisons based on Dunnett's test.

Two-way analysis of variance was used to assess facility differences in the 4 groups in the rate of increase in alveolar bone height, revealing no significant treatment-by-facility interaction (p = 0.795). This suggests that no marked facility differences existed with respect to response.

### 3. Safety evaluation

Major adverse events for which causal relationships with the investigational drug were not ruled out before breaking the blind included positive urinary albumin, increased urinary excretion of β2-microglobulin and N-acetyl-beta-D-glucosamidase, increased serum creatine kinase and C-reactive protein and increased cases of hypersensitive dentine (see Table S4 of supporting items). Frequencies of these adverse events were independent of FGF-2 concentration. No serious adverse events were observed throughout the clinical trial period. A possible association was also considered between frequency of adverse events observed during the trial and the investigational drug administration. None of the adverse events exhibited a strong causal relationship or were severe, and except for one case, all events resolved without any special treatment. For each group, the presence/absence and frequency of adverse events were calculated. Fisher's exact test showed that group allocations exhibited no association to the presence/absence of adverse events (p = 0.469). In addition, during the inspection following FGF-2 administration (Fig. 1), no FGF-2 or anti-FGF-2 antibodies were detected in the serum of any patients.

## Discussion

Originally isolated from bovine hypophysis in the 1970s, FGF-2 is a protein with a molecular weight of 17,000 that acts to promote proliferation of fibroblasts. Researchers have isolated, refined and genetically cloned human FGF-2 to clarify numerous different biological activities of the protein. As yet, many studies have reported that FGF-2 stimulates proliferation of numerous kinds of cells, including not just fibroblasts, but also vascular endothelial, vascular smooth muscle, neuroectodermal, osteoblast, cartilage and epidermal cells. The protein is now known to be deeply involved in cell proliferation and differentiation and also in control of extracellular matrix generation during the processes of tissue generation and wound healing [20]–[25]. Many recent reports in the field of regenerative medicine have described the use of cytokines as “signaling molecules”, stimulating adequate proliferation and differentiation of tissue stem cells. Among those cytokines, FGF-2 is winning attention from researchers due to activity in promoting proliferation of mesenchymal stem cells while maintaining multilineage potential [6]. The protein has already been utilized in a human intractable ulcer-curing drug (Fiblast Spray; Kaken Pharmaceutical Co., Ltd.) for more than 4 years.

We have already studied the stimulation of periodontal tissue regeneration by FGF-2 in animal models and believe that the protein represents a major candidate for a periodontal tissue-regenerating agent. This is based on stimulation of proliferation for both kinds of cell groups that rebuild hard and soft tissues along with strong angiogenic activity, which is indispensable in tissue regeneration. Animal tests have revealed that in artificial models of periodontal tissue defect in beagles [13], [15] and non-human primates (*M. fascicularis*) [14], FGF-2 significantly stimulates neogenesis of alveolar bone, periodontal ligament and cementum, without invoking abnormal effects such as down-growth of the gingival epithelia, resorption of the dental root or ankylosis.

Based on effective concentrations of FGF-2 for periodontal tissue regeneration in animal trials, in addition to the results of our Phase I trial in which FGF-2 was administered intravenously to healthy adult humans, we determined the concentrations and doses administered to periodontal regions of patients in the present clinical trial. More specifically, the results of testing with artificial defect models of periodontal tissue in beagles led us to estimate that an effective FGF-2 concentration for stimulation of periodontal tissue regeneration was 0.03–0.3%. This range of concentrations was therefore applied in the present clinical trial. We selected 200 µL as the dose, considering that this was good enough to work on the defect region of periodontal tissue. In addition, preclinical trial results have suggested that the maximum quantity of administered FGF-2 to enter the circulation in the present trial herein would be around 1.2 mg/body, less than the 30 mg/body for which safety was confirmed in our Phase I trial. The 91 patients screened as subjects and a final total of 80 patients were enrolled as subjects. The patient characteristics were almost similar among groups. However, we understand that we could not perfectly eliminate biasing influences of patient characteristics in this study and a larger scale trial is needed in the future.

In evaluating efficacy, we surveyed 74 cases of 2- or 3-walled intrabony defect that satisfied the selection criteria. To evaluate periodontal tissue regeneration, evaluating fibrous attachment accompanied by neogenesis of alveolar bone and cementum is important. Rate of increase in alveolar bone height observed in close proximity to the dental root was measured as a prespecified primary outcome in this clinical trial.

Use of 0.3% FGF-2 stimulated 58.6% regeneration, which was at least comparable with the previous results within 9 months after regenerative therapy [19], [26], [27]. However, no significant difference was identified between Groups P and H in terms of millimeter increments (p = 0.132). To confirm the efficacy of the investigational drug using more conventional methods, data in both % and millimeter increments were used to calculate sample size for the next late Phase II trial. In addition, the minimum clinically effective dose will need to be assessed and determined in a future clinical study involving more patients.

Interestingly, no significant difference was observed between the 4 groups in the millimeter of CAL regained, with all groups scoring around 2 mm (see Table S2 of supporting items). The CAL regained following periodontal surgery is derived from the sum of epithelial and fibrous attachments. If periodontal tissue regeneration accompanied by neogenesis of the alveolar bone and cementum is stimulated, fibrous attachment reproducing the natural anatomical morphology is achieved. However, the majority of CAL acquisition following conventional periodontal surgery has been shown to be due to epithelial attachment unaccompanied by alveolar bone regeneration [28]–[31]. We have previously conducted an animal study using non-human primates and reported that at the FGF-2 administration site, down-growth of gingival epithelial cells was suppressed to achieve fibrous attachment accompanied by neogenesis of the alveolar bone and cementum [14]. In the present study, no significant differences in CAL regained were seen between Group P (conventional periodontal surgery) and the three FGF-2 groups (Table 8). Based on the results of the above-mentioned preclinical study, we deduce that differences may exist between Group P and the three FGF-2 groups in histological ratio of fibrous and epithelial attachments achieving CAL acquisition. Confirmation of the nature of healing tissue requires histological evaluation in a future study.

PD, BOP, GI, MO, REC and KG are generally used to assess pathology in periodontal disease. These parameters do not directly assess the efficacy of FGF-2 in periodontal tissue regeneration, and were selected in the present study as secondary outcome measures to ascertain whether FGF-2 would cause abnormal periodontal healing following periodontal surgery. The fact that no significant differences among these secondary outcome measures for the 4 groups showed that FGF-2 administration did not cause abnormal healing of periodontium following periodontal surgery. Furthermore, frequency of PD, GI, and BOP all dropped over time in all groups after periodontal surgical treatment. These findings show that we can expect FGF-2 administration to provide a therapeutic process similar to that of the conventional flap operation, in addition to the healing outcome of periodontal tissue regeneration. Yet another observation was the lack of recognisable difference in changes to REC and KG, which accompanies periodontal surgical treatment, between Group P and the other 3 groups receiving FGF-2 administration. This confirms that FGF-2 administration does not cause peculiar gingival recession or reduce keratinized gingiva. PlI offers a parameter for assessing the amount of plaque causing periodontal disease, and since the degree of plaque deposition can affect the prognosis of periodontal surgery, this parameter was also selected as a secondary outcome measure. In this clinical study, no significant intergroup differences were seen in PlI. Moreover, radiography was performed for 67 patients who willingly and positively responded to our “recall” for imaging between week 83 and 132 (inclusive) after administration of investigational drugs (Group P, n = 19; Group L, n = 15; Group M, n = 18; Group H, n = 16). Among these 67 patients, no cases suggested an abnormal increase in alveolar bone exceeding the cementoenamel junction or an equivalent control point or ankylosis (data not shown).

The periodontal ligament comprises heterogenous cell populations and researchers have predicted the existence of some progenitor cells that can differentiate into cementoblasts or osteoblasts [32]–[34]. A recent study reported that some cells within the ligament express STRO-1 and CD146 mesenchymal stem cell markers. Such cells, according to the study, differentiate into cementoblast-like cells, adipocytes and collagen-forming cells. Our previous in vitro studies have clarified that FGF-2 facilitates proliferation while maintaining the differentiation of human periodontal ligament cells (HPDLs). In addition, we now know that the protein does not just stimulate angiogenesis, an action indispensable in the regeneration of tissue, but also increases the production of various types of extracellular matrix from HPDLs [21], [35]–[37]. In short, FGF-2 creates a local environment suitable for the regeneration of periodontal tissue through the activities described above, as part of the mechanism by which regeneration of periodontal tissue is stimulated.

In our clinical trial, to identify adverse events from FGF-2 administered to a particular region of periodontal tissue, we conducted an interview and visual inspection to check the whole body of the patient, checked oral cavity findings and performed clinical inspection. No relationships were identified between administration of the investigational drug and the frequency of any adverse events (see Table S4 of supporting items). Although some adverse events did emerge in patients administered FGF-2, no relationship was recognizable between frequency of these events and FGF-2 concentrations. In addition, the same adverse events also emerged in the placebo group. Those effects are therefore not specific to groups administered FGF-2 and do not appear attributable to the drugs administered. Another reason why we consider that FGF-2 administered locally to periodontal tissue seldom travels through the whole body to create adverse drug reactions is that the protein was undetectable in serum after drug administration. Furthermore, no patients displayed increased levels of anti-FGF-2 antibody after administration, suggesting that FGF-2 is free from antibody production, an adverse drug reaction often seen with other proteinaceous agents. In short, none of the results of this particular clinical trial suggest any clinical problems concerning the safety of administering FGF-2 to patients with periodontitis. One more piece of evidence supporting the high safety of FGF-2 applied locally to periodontal tissue is that this therapy has already been used for more than 5 years as a remedy for intractable ulcers (Fiblast spray).

Periodontitis shortens the life of teeth and can thus reduce QOL in middle-aged to elderly individuals [4]. To maintain and promote oral health, new therapies must be established for safe and efficient regeneration of periodontal tissue. Cytokine therapy has thus been winning attention for the last decade [6]–[15], [19]. However, few double-blinded clinical trials have used multiple facilities in compliance with GCP guidelines to confirm the efficacy of a single cytokine alone as a stimulator of periodontal tissue regeneration. In the present clinical trial, 0.3% FGF-2 improved CAL by about 2 mm at 36 weeks from base. And more importantly, rate of increase in bone height observed in close proximity to the dental root was significantly improved in 0.3% FGF-2 treatment group compared to placebo group at 36 weeks. These findings were clinically interpreted that some efficacy could be expected from FGF-2 in stimulating regeneration of periodontal tissue. Thus, we concluded in this study that FGF-2 therapy can be efficacious in regenerating periodontal tissue. However, an important limitation of this study is the small sample size of the trial. This trial is still preliminary, and several trials need to be performed before FGF-2 drug can be placed on the market. In future, we plan to clarify the efficacy of FGF-2 drug, determine the optimal dose for clinical use and confirm in more detail the safety of FGF-2 in a large Phase II study. And then Phase III will be performed to confirm the efficacy and safety of the invitational drug.

(This clinical trial was conducted at the request of Kaken Pharmaceutical Co., Ltd.)

## Supporting Information

Checklist S1 CONSORT Checklist.
(0.04 MB DOC)

Protocol S1 Trial Protocol.
(0.11 MB DOC)

Table S1 Clinical inspections.
(0.03 MB DOC)

Table S2 Changes in periodontal tissue. Mean and standard deviations are shown. *Data at 36 weeks were missing for 1 patient in Group M.
(0.07 MB DOC)

Table S3 Changes in periodontal tissue. All of data at 36 weeks were missing for 1 patient in Group M. Data for MO were missing at 12 weeks for each patient in Groups P and H, at 24 weeks in Group H and at 36 weeks in Group H.
(0.07 MB DOC)

Table S4 List of adverse drug reactions. *Pains experienced by 1 patient in Group M required therapy, and the patient began to experience pain at the surgical site starting 8 days after administration that resolved 35 days after administration with the use of drugs such as cefcapene pivoxil hydrochloride, lysozyme hydrochloride, rebamipide and loxoprofen sodium.
(0.04 MB DOC)
