# Supplementary material for: Periodontal Tissue Regeneration Using Fibroblast Growth Factor -2: Randomized Controlled Phase II Clinical Trial
Source: PLoS One. 2008 Jul 2;3(7):e2611. doi: 10.1371/journal.pone.0002611 (PMC2432040; doi:10.1371/journal.pone.0002611)
Supplement: Protocol S1 — Trial Protocol. (0.11 MB DOC) [file pone.0002611.s002.doc]

# STUDY PROTOCOL

**Trial in Periodontal Tissue Regeneration Using Fibroblast Growth Factor –2: Randomized Controlled Phase II Clinical Trial**

This was translated from Japanese original protocol, which was created on 22^nd^ November 2001.

**TABLE OF CONTENTS**

**1.0 INTRODUCTION**

**2.0 STUDY OBJECTIVES**

**3.0 TRIAL DESIGN**

**3.1 Main outcome measures**

**3.2 Study Design**

**3.3 Measures to Minimize bias**

**3.4 Study Product**

**3.4.1 Physicochemical and Pharmaceutical Properties and Formulation Composition**

**3.5 Duration of the Study**

**4.0 SELECTION OP PARTICIPANTS**

**5.0 STUDY PRODUCTS AND TREATMENT**

**5.1 Products**

**5.2 Treatment**

**6.0 EFFICACIY AND SAFETY ASSESSMENT**

**6.1 Efficacy assessment**

**6.1.1 Standardized radiography for regions of investigation**

**6.1.2 Inspection of periodontal tissue around the tooth under investigation**

**6.2 Safety assessment**

**6.2.1 Observation of subjective symptoms and objective findings**

**6.2.2 Clinical inspections**

**6.2.3 Measurement of anti-FGF-2 antibody levels in serum**

**6.2.4 Measurement of FGF-2 concentration within serum**

**7.0 ADVERSE EVENTS**

**7.1 Definition**

**7.2 Evaluation of Adverse Events**

**8.0 STATISTICAL SUMMARY**

**9.0 ETHICS AND RESEARCH INTEGRITY**

**9.1 Fundamental principle**

**9.2 Institutional Review Board**

**9.3 Ensuring Human-rights**

**10.0 REFERENCES**

**1.0 INTRODUCTION**

Discovery of FGF-2 has its origin in Gospodarowz's^1^ discovery of a protein that accelerates proliferation of fibroblasts from bovine pituitary gland and in 1974 he named it FGF. In 1984, it was clarified^2,3^ that FGF consists of two proteins, a basic fibroblast growth factor (FGF-2) and acidic fibroblast growth factor (FGF-1) having an isoelectric point in basic and acidic regions respectively.

It was clarified that in vitro FGF-2 accelerates not only proliferation of fibroblasts but also the proliferation, streaming and differentiation of a variety of cells such as vascular endothelial cells, vascular smooth muscle cells and epithelial cells^4,5^. It was also reported that in vivo FGF-2 accelerates angiogenesis and granulation tissue formation^4,6,7,8^ and it is distributed ubiquitously to most of the intravital tissues^12^. Based on these effects, it was expected that FGF-2 activates and accelerates a variety of processes in wound healing. In fact, the protein has already been utilized in a human intractable ulcer-curing drug (Fiblast Spray; Kaken Pharmaceutical Co., Ltd.) for more than 4 years. On the other hand, many recent reports in the field of regenerative medicine have described the use of cytokines as “signaling molecules”, inducing adequate proliferation and differentiation of what are known as tissue stem cells. Among those cytokines, FGF-2 is winning attention from researchers due to activity in promoting proliferation of mesenchymal stem cells while maintaining the multipotent nature of such cells.

All over the world, periodontitis remains highly prevalent, can lead loss of the affected teeth, and thus threaten quality of life for middle-aged population as far as oral functions are concerned. Unfortunately, no conventional periodontal treatments can, at best, heal the scar in the affected region and ever regenerate lost periodontal tissue or normal structure and functionality. Considering that the “mouth” and “teeth” have various aesthetic and functional roles to play, establishing a brand-new treatment that enables the regeneration of periodontal tissue is very important. Recently, researchers confirmed the existence of mesenchymal stem cells within the periodontal ligament, one of the components of periodontal tissue. We studied induction of periodontal tissue regeneration by FGF-2 in animal models and found that topical application of FGF-2 induced periodontal tissue regeneration in models of artificial loss of periodontal tissue in beagles and non-human primates. This suggests that FGF-2 may be efficacious in periodontal tissue regeneration of periodontitis patients.

In the phase I clinical trial for 24 healthy male volunteers by single administration of FGF-2 aqueous solution (1mg, 3mg, 10mg, 30 mg), neither adverse reactions nor rise of anti-KCB-1 antibody were observed.

**2.0 STUDY OBJECTIVES**

The purpose of this clinical trial is to both clarify the activity of FGF-2 to regenerate periodontal tissue in periodontitis patients and to confirm drug safety.

**3.0 TRIAL DESIGN**

**3.1 Main outcome measures**

The main outcome measures in the study protocol were: rate of increase in alveolar bone height and millimeter of CAL regained. In addition, we examined whether and to what extent adverse events, for which causal relationships with the investigational drug were not ruled out before breaking the blind, emerged. We set rate of increase in alveolar bone height as the most statistically important outcome (primary outcome).

**3.2 Study Design**

**This is a randomized double-blinded clinical trial of dose responses including placebo comparison, involving 13 dental facilities in compliance with good clinical practice guidelines. This trial is on the early Phase II stage. The clinical trial is conducted according to the schedule shown below.**

| Item | Before registration | Before administration | Administration | After administration | | | | | | | | | | | |
| --- | --- | --- | --- | --- | --- | --- | --- | --- | --- | --- | --- | --- | --- | --- | --- |
|  |  |  |  | Hour | | | | Date | | Week | | | | | |
|  |  |  |  | 1 | 2 | 4 | 24 | 2 | 3 | 1 | 2 | 4 | 12 | 24 | 36 |
| Patient agreement | ● |  |  |  |  |  |  |  |  |  |  |  |  |  |  |
| Case registration | ● |  |  |  |  |  |  |  |  |  |  |  |  |  |  |
| Patient background | ● | ● |  |  |  |  |  |  |  |  |  |  |  |  |  |
| Administration of investigational drug |  |  | ● |  |  |  |  |  |  |  |  |  |  |  |  |
| Radiography | | ● |  |  |  |  |  |  |  |  |  |  | ● | ● | ● |
| Clinical attachment level | | ● |  |  |  |  |  |  |  |  |  |  | ● | ● | ● |
| Periodontal tissue inspections | | ● |  |  |  |  |  |  |  |  |  |  | ● | ● | ● |
| Subjective symptoms and objective findings |  | ● |  |  |  |  | ● | | | ● | ● | ● | ● | ● | ● |
| Clinical inspections | ● | ● |  |  |  |  |  |  |  | ● | ● | ● |  |  |  |
| Oral cavity inspection | ● |  |  |  |  |  |  |  |  |  |  |  |  |  |  |
| Anti-FGF-2 antibody in serum |  | ● |  |  |  |  |  |  |  |  | ● | ● |  |  |  |
| FGF-2 concentration in serum  (n=6 from each group) | | ● |  | ● | ● | ● | ● |  |  |  |  |  |  |  |  |

**3.3 Measures to Minimize bias**

We will install an independent organization, the Registration Center, to keep treatment allocation inaccessible to any patients or other individuals involved in the trial. The Registration Center will create an allocation table where a block size of 4 cases per block is allocated to investigational drugs comprising placebo (Group P), 0.03% FGF-2 (Group L), 0.1% FGF-2 (Group M) or 0.3% FGF-2 (Group H). According to this allocation table, a label indicating the corresponding drug number will be attached to each and every vial of drug. After drugs are allocated, the Registration Center will seal and keep the allocation table in confidence until the clinical trial is over. Freeze-dried drugs for Groups P, L, M and H must be created indistinguishable based on appearance.

Investigators at each facility will check all inclusion and exclusion criteria and register patients one at a time by sending patient information that will be obtained under informed consent to the Registration Center. The center again will check the documents to make sure that each subject satisfied all inclusion and exclusion criteria, and randomly allocate subjects as necessary to receive drugs based on a single block consisting of one drug sample each from Groups P, L, M, and H, and then fax back the assigned drug numbers to the investigators. The blind will not be broken until this clinical trial is completely finished.

**3.4 Study Product**

**3.4.1 Physicochemical and pharmaceutical properties and formulation composition**

1) Development code: KCB-1, clinical trial code: KCB-1D

2) Generic name <JAN, INN>: trafermin (genetic recombinant)

3) Chemical name or entity: proteins consisting of 154 and 153 amino acid residues (N-terminal; Ala-:Ala: not less than 65%, Ala: not more than 35%) manufactured as a recombinant by expression of human genomic gene

4) Molecular formula, molecular weight;

Molecular formula;

C764H1201N217O219S6 (a protein consisting of 154 amino acids)

C761H1196N216O218S6 (a protein consisting of 153 amino acids)

Molecular weight;

17,122.67(a protein consisting of 154 amino acids)

17,051.59(a protein consisting of 153 amino acids)

5) Dosage form: lyophilized formulation (liquid for reconstitution, hydroxypropyl -cellulose Liquid, is attached)

6) Contents of formulation: KCB-1D lyophilized formulations

0.03 % formulation: one vial contains 300 μg of trafermin

0.1 % formulation: one vial contains 1000 μg of trafermin

0.3 % formulation: one vial contains 3000 μg of trafermin

HPC Liquid (attached liquid for reconstitution:): 3% hydroxypropylcellulose solution

**3.5 Duration of the Study**

This clinical trial including recruitment of subjects will be performed from December 1, 2001 to November 30, 2003.

**4.0 SELECTION OP PARTICIPANTS**

Patients with periodontitis visiting any of the 13 dental institutions listed below will be requested to participate.

| Trial facilities | Investigators |
| --- | --- |
| Dental Hospital, Health Sciences University of Hokkaido | Yusuke Kowashi |
| Medical and Dental Clinic, Health Sciences University of Hokkaido | Takeo Fujii |
| Tohoku University Dental Hospital | Hidetoshi Shimauchi |
| Aichigakuin University Dental Hospital | Mitsuo Fukuda |
| Asahi University Dental Hospital | Toshiaki Shibutani |
| Osaka University Dental Hospital | Masahiro Kitamura |
| Okayama University Hospital of Dentistry | Shogo Takashiba |
| Hiroshima University Hospital of Dentistry | Hidemi Kurihara |
| Tokushima University Dental Hospital | Jun-ichi Kido |
| Kyushu University Dental Hospital | Takafumi Hamachi |
| Nagasaki University Hospital Attached School of Dentistry | Yoshitaka Hara |
| Kagoshima University Dental Hospital | Yuichi Izumi |
| Fukuoka Dental College Hospital | Takao Hirofuji |

In compliance with Good Clinical Practice guidelines, prospective patients who provide written informed consent will undergo clinical inspection and an oral cavity diagnosis. We will then select as subjects 80 patients who satisfy the selection and exclusion criteria described below.

| We selected those patients who met the criteria listed below, from those who the investigators and/or sub-investigators determined were in need of flap operation. | |
| --- | --- |
| 1) | Those diagnosed as having, from radiography and other results, 2- or 3-walled vertical intrabony defect ≥3 mm deep from the top of the remaining alveolar bone |
| 2) | Those who have accomplished initial preparation and have been showing good compliance |
| 3) | Those with mobility of the tooth to investigate of Degree 2 or less and with width of attached gingiva for which the existing Guided Tissue Regeneration (GTR) treatment is considered appropriate |
| 4) | Those for whom supportive periodontal treatment (SPT) is applicable, in accordance with usual post-operative procedures following flap operation and GTR treatment |
| 5) | Those whose oral hygiene is well established and who are able to perform appropriate tooth brushing following instructions of the investigators and/or sub-investigators after investigational drug administration |
| 6) | Those ≥20-years-old and <65-years-old |
| 7) | Those who understand the purposes of the trial and are capable of making an independent decision to comply with trial requirements |
| 8) | Those who are able to visit their hospitals in accordance with the trial schedule |

Criteria for selecting subjects

| 1) | Those administered a calcium antagonist during the 4 weeks preceding administration of the investigational drug |
| --- | --- |
| 2) | Those in need of administration of adrenal cortical steroid (equivalent to>20 mg/day of Predonin) within 4 weeks after investigational drug administration |
| 3) | Those scheduled to undergo a surgical operation in the vicinity of the tooth to investigate within 36 weeks after investigational drug administration |
| 4) | Those with coexisting mental or consciousness disorder |
| 5) | Those with coexisting malignant tumour or history of the same |
| 6) | Those with coexisting diabetes (HbA_1C_ >6.5%) |
| 7) | Those in an extremely poor nutritional condition (serum albumin concentration <2 g/dL) |
| 8) | Those with ≥200 mL of blood drawn during the 4 weeks preceding investigational drug administration |
| 9) | Those administered another investigational drug during the 24 h preceding investigational drug administration |
| 10) | Those with coexisting disorder of the kidney, liver, blood and/or circulatory system (Grade 2 or above) |
| 11) | Those who are either pregnant, possibly pregnant or breast-feeding, or who hope to become pregnant during the period of the trial |
| 12) | Those with a previous history of hypersensitivity to a protein drug |
| 13) | Others who the investigators or sub-investigators determined as unsuitable for the trial |

**Criteria for excluding subjects**

**5.0 STUDY PRODUCTS AND TREATMENT**

**5.1 Products**

This trial will employ recombinant human FGF-2 (Code No. KCB-1; Kaken Pharmaceutical Co., Ltd., Tokyo, Japan) produced by genetic recombination which introduce into *Escherichia coli* the gene for human FGF-2. To improve operability of drug administration to the region of alveolar bone defect, before administration we will mix freeze-dried FGF-2 with 3% hydroxypropylcellulose, a colorless and viscid solution, and prepare a gel-like investigational drug of this clinical trial (Code No. KCB-1D). FGF-2 concentration in the investigational drug will be then adjusted to 0% (placebo), 0.03%, 0.1% or 0.3%, and administered to the region of investigation within 2 h of adjustment. Before the start and after completion of investigational drug administration, a third-party organization will measure FGF-2 concentrations for each group to ascertain that FGF-2 concentrations in vials are accurate according to good manufacturing practice (GMP) standards.

**5.2 Treatment**

Surgical treatment will be performed in the region of investigation, during which time 200 μL of investigational drug will be administered to the bone defect region. Each subject will have received a standard initial preparation, including oral hygiene instruction, full-mouth scaling, and root planning before surgical treatment in order to minimize bacterial insult and reduce variability between lesions at baseline.

**6.0 EFFICACIY AND SAFETY ASSESSMENT**

**6.1 Efficacy assessment**

The main outcome measures in the study protocol are rate of increase in alveolar bone height and millimeter of CAL regained. In addition, we will examine whether and to what extent adverse events, for which causal relationships with the investigational drug are not ruled out before breaking the blind, will emerge. We set rate of increase in alveolar bone height as the most statistically important outcome (primary outcome). Probing depth (PD), Bleeding on probing (BOP), Gingival index (GI), Mobility of tooth (MO), Recession of gingival (REC) and Width of keratinized gingival (KG) are generally used to assess pathology in periodontal disease. These parameters do not directly assess the efficacy of FGF-2 in periodontal tissue regeneration, and are selected in the present study as secondary outcome measures to ascertain whether FGF-2 would cause abnormal periodontal healing following periodontal surgery. Furthermore, Plaque index (PlI) offers a parameter for assessing the amount of plaque causing periodontal disease, and since the degree of plaque deposition can affect the prognosis of periodontal surgery, this parameter was selected as a secondary outcome measure.

**6.1.1 Standardized radiography for regions of investigation**

Our geometrically standardized radiography employs dental film (Kodak InSight Super Poly-Soft; Eastman Kodak Company, New York, USA) and photograph indicators (Cone Indicator-II; Hanshin Technical Laboratory, Hyogo, Japan) customized with resin stents. Five doctors specializing in dental radiology from the Department of Oral Diagnosis at Tohoku University Graduate School of Dentistry will measure rate of increase in alveolar bone height (%) using the methods described below.

Points A, B, C and D represent the cementoenamel junction, apex, remaining alveolar bone crest and bottom of the bone defect, respectively. The Examiner will measure tooth axis heights between Points A and B, Points A and C, and Points A and D on the X-ray for each patient. To adjust for slight errors due to imaging, measurements for 5 examiners will be multiplied by A-B ratio of before to after administration to correct A-B, A-C and A-D after administration (adjusted A-B, A-C and A-D, respectively). Based on A-D and A-C before administration, C-D will be calculated to serve as an indicator of the depth of the alveolar bone defect.

The rate of increase in alveolar bone will be calculated by dividing [(A-D before administration) - (adjusted A-D after administration)] by C-D before administration.

A

B

C

D

Each of the 5 examiners independently will measure this rate in a blinded measurement and the median of 5 measurements taken from the same image will be then selected for efficacy analysis.

**6.1.2 Inspection of periodontal tissue around the tooth under investigation**

We will measure the items shown below at 6 positions (mesiobuccal, buccal, distobuccal, mediolingual, lingual, distolingual) around each tooth under investigation.

| 1) | Clinical attachment level (CAL): A stent was prepared for each subject. Using as the control point the cementoenamel junction or margin of the restorative material, distance between the control point and bottom of the gingival sulcus was measured for each test subject, using the same periodontal probe. |
| --- | --- |
| 2) | Probing depth (PD): Simultaneously with CAL measurement, we measured distance from the gingival margin to the bottom of the gingival sulcus for each subject using the same periodontal probe |
| 3) | Bleeding on probing (BOP; + or -): The presence of bleeding was checked 10 s after probing. |
| 4) | Gingival index (GI): GI was determined as described by Löe and Silness.^16^ |
| 5) | Mobility of tooth (MO): MO was determined as described by Miller.^17^ |
| 6) | Recession of gingiva (REC): Using as the control point the cementoenamel junction or margin of the restorative material, distance between the control point and gingival margin was measured for each subject, using the same periodontal probe. |
| 7) | Plaque index (PlI): PlI was determined as described by Silness and Löe.^18^ |
| 8) | Width of keratinised gingiva (KG): The shortest distance between the coronal gingival margin and mucogingival junction was measured for each subject, using the same periodontal probe. |

**6.2 Safety assessment**

**6.2.1 Observation of subjective symptoms and objective findings**

Medical findings for both the oral cavity and whole body will be confirmed by interview and visual inspection.

**6.2.2 Clinical inspections**

The inspection items measured and evaluated are shown below. In cases where we discover unusual changes in any of the clinical inspection values listed within 4 weeks after administration of the investigational drug, a follow-up survey will be conducted.

| 1) | Haematological test (2 ml of blood)  Red blood cell count, white blood cell count, haemoglobin, haematocrit, platelet count, differential counts of leukocytes (neutrophils, eosinophils, basophils, lymphocytes, monocytes) |
| --- | --- |
| 2) | Biochemical blood test (2 ml of serum)  Total protein, albumin, blood urea nitrogen, creatinine, uric acid, total cholesterol, total bilirubin, aspartate aminotransferase, alanine aminotransferase, alkaline phosphatase, lactic dehydrogenase, C-reactive protein (CRP), creatine kinase (CK), Na, K, Cl |
| 3) | Urinalysis  Qualitative (10 ml of urine): protein, sugar, urobilinogen  Quantitative (3.5 ml of urine): urinary albumin (in creatinine equivalents), N-acetyl-beta-D-glucosamidase (NAG), beta2-microglobulin |
| 4) | Blood glucose control index test (2 ml of blood): Conducted only before registration  Haemoglobin A_1C_ |
| 5) | Pregnancy test (1 ml of urine): Conducted only before registration  Chorionic gonadotropin |

**6.2.3 Measurement of anti-FGF-2 antibody levels in serum**

The Pharmacokinetics Department of Kaken Pharmaceutical Co., Ltd. will measure levels of anti-FGF-2 antibody (IgG) in serum using ELISA.

**6.2.4 Measurement of FGF-2 concentration within serum**

The Metabolism Research Department of Kaken Pharmaceutical Co., Ltd. will measure FGF-2 concentrations in serum using ELISA.

**7.0 ADVERSE EVENTS**

**7.1 Definition**

An adverse event is any unfavorable and unintended sign (including an abnormal laboratory finding, for example), symptom, or disease temporally associated with the use of the investigational drug, whether or not considered related to the investigational drug.

**7.2 Evaluation of Adverse Events**

The investigators must report any adverse event with the exact name, the onset date, the severity, the provided treatment and the date of disappearance.

Each adverse event should be graded for severity using the following scale and the criteria notified by the Ministry of Health and Welfare of Japanese Government on June 29th, 1998.

1.Mild: The subject can continue the subsequent schedule without any treatment.

2.Moderate: The subject can continue the subsequent schedule with treatments.

3.Severe: The subject cannot continue the subsequent schedule.

The investigators must determine the relationship of the adverse event to the investigational drug.

The investigators must determine whether or not the adverse event is classified as a serious adverse event. A serious adverse event is defined as any experience that suggests a significant hazard, contraindication, side effect, or precaution. A serious adverse event includes any adverse experience that results in any of the following outcomes:

1. Death

2. A life-threatening adverse experience

3. Impatient hospitalization or prolongation of existing hospitalization

4. A disability/incapacity that may interfere with the subject's daily life

5. An adverse experience leading to a disability/incapacity

6. A serious adverse experience at the same level as the above conditions

7. A congenital anomaly/birth

**8.0 STATISTICAL SUMMARY**

For analysis, we will employ SAS version 8.2 software (SAS Institute Inc., Carey, North Carolina, USA). Level of statistical significance will be set at p<0.05 in advance. Data analysis will cover those patients administered the randomly allocated investigational drugs. Analysis will be performed by full analysis set. Additionally, per protocol set analysis will be done for the primary outcome. Between-group comparisons of categorical variables will be performed using Kruskal-Wallis’s test or Fisher’s exact test. To statistically compare the 3 dose groups in terms of % increase in alveolar bone mass with the placebo group, the Dunnett option will be used based on the Mixed procedure in the SAS system, in which adjusted p-values will be computed for multiple comparisons, and analysis for rates of increase during follow-up will be carried out using a repeated-measures analysis of variance with the Mixed procedure.

**9.0 ETHICS AND RESEARCH INTEGRITY**

**9.1 Fundamental principle**

We will perform this trial in complying with ICH Harmonised Tripartite Guideline E6.

**9.2 Institutional Review Board**

Prior to this clinical trial, the study must be approved in writing by the Institutional Review Board at each facility. The facilities will obtain written reapproval of the research by the IRBs at least annually.

**9.3 Ensuring Human-rights**

We comply with Declaration of Helsinki and place maximum priority on ensuring human-rights, welfare and safety of the subjects.

**10.0 REFERENCES**

(1) Gospodarowicz D: Localisation of a fibroblast growth factor and its effect alone and with hydrocortisone on 3T3 cell growth. Nature, 249: 123, 1974.

(2) Böhlen P, et al: Isolation and partial molecular characterization of pituitary fibroblast growth factor. Proc Natl Acad Sci USA, 81: 5364, 1984.

(3) Thomas KA, et al: Purification and characterization of acidic fibroblast growth factor from bovine brain. Proc Natl Acad Sci USA, 81: 357, 1984.

(4) Gospodarowicz D, et al: Structural characterization and biological functions of fibroblast growth factor. Endocrine Reviews, 8(2): 95, 1987.

(5) O’Keefe EJ, et al: Stimulation of growth of keratinocytes by basic fibroblast growth factor. J Invest Dermatol, 90: 767, 1988.

(6) Sporn MB. Eds: Peptide growth factors and their receptorsⅠ. 369, 1991.

(7) Folkman J, et al: Angiogenic factors. Science, 235: 442, 1987.

(8) Montesano R, et al: Basic fibroblast growth factor induces angiogenesis in vitro. Proc Natl Acad Sci USA, 83: 7297-7301, 1986.

(9) Baird A, et al: Molecular characterization of fibroblast growth factor: distribution and biological activities in various tissues. Rec Prog Horm Res, 42: 143, 1986.

(10) Löe H, et al: Periodontal disease in pregnancy. I. Prevalence and severity. Acta Odontol Scand, 21: 533, 1963.

(11) Miller SC, et al: Textbook of Periodontia, 3rd ed. Philadelphia and Toronto: The Blakiston Co., 1950.

(12) Silness J, et al: Periodontal disease in pregnancy. II. Correlation between oral hygiene and periodontal condition. Acta Odontol Scand, 22: 121, 1964.
