# Supplementary material for: Periodontal Tissue Regeneration Using Fibroblast Growth Factor -2: Randomized Controlled Phase II Clinical Trial
Source: PLoS One. 2008 Jul 2;3(7):e2611. doi: 10.1371/journal.pone.0002611 (PMC2432040; doi:10.1371/journal.pone.0002611)
Supplement: Table S1 — Clinical inspections. (0.03 MB DOC) [file pone.0002611.s003.doc]

***Table S1:* Clinical inspections**

| 1) | Haematological test (2 ml of blood)  Red blood cell count, white blood cell count, haemoglobin, haematocrit, platelet count, differential counts of leukocytes (neutrophils, eosinophils, basophils, lymphocytes, monocytes) |
| --- | --- |
| 2) | Biochemical blood test (2 ml of blood serum)  Total protein, albumin, blood urea nitrogen, creatinine, uric acid, total cholesterol, total bilirubin, aspartate aminotransferase, alanine aminotransferase, alkaline phosphatase, lactic dehydrogenase, C-reactive protein, creatine kinase, Na, K, Cl |
| 3) | Urinalysis  Qualitative (10 ml of urine): protein, sugar, urobilinogen  Quantitative (3.5 ml of urine): urinary albumin (in creatinine equivalents), N-acetyl-beta-D-glucosamidase, beta2-microglobulin |
| 4) | Blood glucose control index test (2 ml of blood): Conducted only before registration  Haemoglobin A_1C_ |
| 5) | Pregnancy test (1 ml of urine): Conducted only before registration  Chorionic gonadotropin |
