# Supplementary material for: Periodontal Tissue Regeneration Using Fibroblast Growth Factor -2: Randomized Controlled Phase II Clinical Trial
Source: PLoS One. 2008 Jul 2;3(7):e2611. doi: 10.1371/journal.pone.0002611 (PMC2432040; doi:10.1371/journal.pone.0002611)
Supplement: Table S2 — Changes in periodontal tissue. Mean and standard deviations are shown. *Data at 36 weeks were missing for 1 patient in Group M. (0.07 MB DOC) [file pone.0002611.s004.doc]

***Table S2:* Changes in periodontal tissue**

|  | Group P  (n=19) | | | | Group L  (n=19) | | | | Group M  (n=19) | | | | Group H  (n=17) | | | |
| --- | --- | --- | --- | --- | --- | --- | --- | --- | --- | --- | --- | --- | --- | --- | --- | --- |
| CAL (mm) |  | | | |  | | | |  | | | |  | | | |
| Before operation | 9.11 | ( | 2.16 | ) | 8.37 | ( | 2.73 | ) | 8.32 | ( | 2.87 | ) | 8.41 | ( | 2.94 | ) |
| 12 weeks | 7.16 | ( | 2.69 | ) | 7.53 | ( | 2.14 | ) | 6.76 | ( | 2.95 | ) | 6.26 | ( | 3.15 | ) |
| 24 weeks | 6.84 | ( | 2.27 | ) | 6.92 | ( | 2.73 | ) | 6.58 | ( | 3.06 | ) | 6.06 | ( | 3.05 | ) |
| 36 weeks* | 6.47 | ( | 2.20 | ) | 6.37 | ( | 2.81 | ) | 5.97 | ( | 2.76 | ) | 6.24 | ( | 3.21 | ) |
| CAL regained (mm) |  | | | |  | | | |  | | | |  | | | |
| 12 weeks | 1.95 | ( | -1.51 | ) | 0.84 | ( | 2.03 | ) | 1.55 | ( | 2.36 | ) | 2.15 | ( | 0.90 | ) |
| 24 weeks | 2.26 | ( | 1.56 | ) | 1.45 | ( | 1.96 | ) | 1.74 | ( | 2.42 | ) | 2.35 | ( | 1.17 | ) |
| 36 weeks* | 2.63 | ( | 1.54 | ) | 2.00 | ( | 2.08 | ) | 2.14 | ( | 2.08 | ) | 2.18 | ( | 1.33 | ) |
| % of CAL regained |  | | | |  | | | |  | | | |  | | | |
| 12 weeks | 23.22 | ( | 18.95 | ) | 7.01 | ( | 24.75 | ) | 15.89 | ( | 36.83 | ) | 28.73 | ( | 16.65 | ) |
| 24 weeks | 25.50 | ( | 18.10 | ) | 16.03 | ( | 28.49 | ) | 18.68 | ( | 38.53 | ) | 31.22 | ( | 20.11 | ) |
| 36 weeks* | 29.65 | ( | 17.00 | ) | 24.03 | ( | 25.31 | ) | 25.54 | ( | 28.45 | ) | 29.69 | ( | 23.14 | ) |
| PD (mm) |  | | | |  | | | |  | | | |  | | | |
| Before operation | 5.63 | ( | 1.21 | ) | 5.42 | ( | 1.57 | ) | 5.05 | ( | 2.07 | ) | 5.94 | ( | 1.68 | ) |
| 12 weeks | 3.05 | ( | 1.13 | ) | 3.05 | ( | 1.22 | ) | 2.74 | ( | 1.19 | ) | 2.71 | ( | 1.26 | ) |
| 24 weeks | 2.74 | ( | 0.93 | ) | 3.05 | ( | 1.27 | ) | 2.63 | ( | 1.12 | ) | 2.29 | ( | 1.21 | ) |
| 36 weeks* | 2.53 | ( | 0.77 | ) | 2.79 | ( | 1.13 | ) | 2.50 | ( | 1.10 | ) | 2.41 | ( | 1.37 | ) |
| REC (mm) |  | | | |  | | | |  | | | |  | | | |
| Before operation | 2.37 | ( | 1.83 | ) | 2.05 | ( | 1.50 | ) | 2.00 | ( | 2.29 | ) | 1.76 | ( | 1.60 | ) |
| 12 weeks | 3.08 | ( | 1.93 | ) | 3.16 | ( | 2.03 | ) | 2.66 | ( | 1.94 | ) | 2.53 | ( | 1.66 | ) |
| 24 weeks | 3.03 | ( | 1.70 | ) | 2.74 | ( | 2.02 | ) | 2.47 | ( | 2.12 | ) | 2.71 | ( | 2.08 | ) |
| 36 weeks* | 2.58 | ( | 1.57 | ) | 2.68 | ( | 2.19 | ) | 2.31 | ( | 2.12 | ) | 2.59 | ( | 2.12 | ) |
| KG (mm) |  | | | |  | | | |  | | | |  | | | |
| Before operation | 4.95 | ( | 2.17 | ) | 4.29 | ( | 1.94 | ) | 4.55 | ( | 2.30 | ) | 4.71 | ( | 2.39 | ) |
| 12 weeks | 3.95 | ( | 1.51 | ) | 3.84 | ( | 2.22 | ) | 3.71 | ( | 2.17 | ) | 4.12 | ( | 2.20 | ) |
| 24 weeks | 3.68 | ( | 1.60 | ) | 4.00 | ( | 2.24 | ) | 3.92 | ( | 2.53 | ) | 4.12 | ( | 2.20 | ) |
| 36 weeks* | 3.84 | ( | 1.61 | ) | 4.16 | ( | 2.32 | ) | 3.92 | ( | 2.39 | ) | 3.91 | ( | 2.18 | ) |

Mean and standard deviations are shown. *Data at 36 weeks were missing for 1 patient in Group M.
