# Supplementary material for: Periodontal Tissue Regeneration Using Fibroblast Growth Factor -2: Randomized Controlled Phase II Clinical Trial
Source: PLoS One. 2008 Jul 2;3(7):e2611. doi: 10.1371/journal.pone.0002611 (PMC2432040; doi:10.1371/journal.pone.0002611)
Supplement: Table S3 — Changes in periodontal tissue. All of data at 36 weeks were missing for 1 patient in Group M. Data for MO were missing at 12 weeks for each patient in Groups P and H, at 24 weeks in Group H and at 36 weeks in Group H. (0.07 MB DOC) [file pone.0002611.s005.doc]

***Table S3:*** **Changes in periodontal tissue**

|  |  | Group P  (n=19) | | | |  | Group L  (n=19) | | | |  | Group M  (n=19) | | | |  | Group H  (n=17) | | | |
| --- | --- | --- | --- | --- | --- | --- | --- | --- | --- | --- | --- | --- | --- | --- | --- | --- | --- | --- | --- | --- |
| GI | (Score) | (0) | (1) | (2) | (3) |  | (0) | (1) | (2) | (3) |  | (0) | (1) | (2) | (3) |  | (0) | (1) | (2) | (3) |
| Number  of  patients | Before operation | 7 | 5 | 7 | 0 |  | 4 | 9 | 6 | 0 |  | 5 | 7 | 7 | 0 |  | 2 | 7 | 8 | 0 |
|  | 12 weeks | 9 | 5 | 5 | 0 |  | 9 | 6 | 4 | 0 |  | 14 | 3 | 2 | 0 |  | 10 | 6 | 1 | 0 |
|  | 24 weeks | 14 | 3 | 2 | 0 |  | 14 | 4 | 1 | 0 |  | 12 | 6 | 1 | 0 |  | 12 | 4 | 1 | 0 |
|  | 36 weeks | 11 | 5 | 3 | 0 |  | 14 | 4 | 1 | 0 |  | 13 | 3 | 2 | 0 |  | 12 | 4 | 1 | 0 |
| MO | (Score) | (0) | (1) | (2) | (3) |  | (0) | (1) | (2) | (3) |  | (0) | (1) | (2) | (3) |  | (0) | (1) | (2) | (3) |
| Number  of  patients | Before operation | 12 | 7 | 0 | 0 |  | 11 | 7 | 1 | 0 |  | 10 | 9 | 0 | 0 |  | 8 | 9 | 0 | 0 |
|  | 12 weeks | 12 | 6 | 0 | 0 |  | 12 | 7 | 0 | 0 |  | 13 | 6 | 0 | 0 |  | 10 | 5 | 1 | 0 |
|  | 24 weeks | 11 | 8 | 0 | 0 |  | 13 | 6 | 0 | 0 |  | 15 | 4 | 0 | 0 |  | 8 | 8 | 0 | 0 |
|  | 36 weeks | 13 | 6 | 0 | 0 |  | 12 | 7 | 0 | 0 |  | 15 | 3 | 0 | 0 |  | 8 | 8 | 0 | 0 |
| PlI | (Score) | (0) | (1) | (2) | (3) |  | (0) | (1) | (2) | (3) |  | (0) | (1) | (2) | (3) |  | (0) | (1) | (2) | (3) |
| Number  of  patients | Before operation | 9 | 7 | 3 | 0 |  | 8 | 11 | 0 | 0 |  | 15 | 4 | 0 | 0 |  | 10 | 6 | 1 | 0 |
|  | 12 weeks | 9 | 8 | 2 | 0 |  | 9 | 7 | 3 | 0 |  | 14 | 5 | 0 | 0 |  | 8 | 8 | 1 | 0 |
|  | 24 weeks | 12 | 6 | 0 | 1 |  | 14 | 5 | 0 | 0 |  | 14 | 5 | 0 | 0 |  | 6 | 11 | 0 | 0 |
|  | 36 weeks | 9 | 9 | 0 | 1 |  | 14 | 4 | 1 | 0 |  | 12 | 6 | 0 | 0 |  | 11 | 5 | 1 | 0 |
| BOP | (Score) | (-) | | (+) | |  | (-) | | (+) | |  | (-) | | (+) | |  | (-) | | (+) | |
| Number  of  patients | Before operation | 2 | | 17 | |  | 3 | | 16 | |  | 3 | | 16 | |  | 1 | | 16 | |
|  | 12 weeks | 8 | | 11 | |  | 10 | | 9 | |  | 13 | | 6 | |  | 9 | | 8 | |
|  | 24 weeks | 14 | | 5 | |  | 10 | | 9 | |  | 12 | | 7 | |  | 13 | | 4 | |
|  | 36 weeks | 14 | | 5 | |  | 13 | | 6 | |  | 15 | | 3 | |  | 14 | | 3 | |

All of data at 36 weeks were missing for 1 patient in Group M. Data for MO were missing at 12 weeks for each patient in Groups P and H, at 24 weeks in Group H and at 36 weeks in Group H.
