# Supplementary material for: Periodontal Tissue Regeneration Using Fibroblast Growth Factor -2: Randomized Controlled Phase II Clinical Trial
Source: PLoS One. 2008 Jul 2;3(7):e2611. doi: 10.1371/journal.pone.0002611 (PMC2432040; doi:10.1371/journal.pone.0002611)
Supplement: Table S4 — List of adverse drug reactions. *Pains experienced by 1 patient in Group M required therapy, and the patient began to experience pain at the surgical site starting 8 days after administration that resolved 35 days after administration with the use of drugs such as cefcapene pivoxil hydrochloride, lysozyme hydrochloride, rebamipide and loxoprofen sodium. (0.04 MB DOC) [file pone.0002611.s006.doc]

***Table S4:* List of adverse drug reactions**

|  | Group P | Group L | Group M | Group H |
| --- | --- | --- | --- | --- |
| Number of patients | 20 | 19 | 20 | 20 |
| Decrease in white blood cell count |  | 1 | 1 | 1 |
| Increase in red blood cell count | 1 | 1 |  |  |
| Decrease in percentage of neutrophils | 1 |  | 1 |  |
| Increase in percentage of neutrophils |  | 1 |  |  |
| Decrease in percentage of lymphocytes |  | 1 |  |  |
| Increase in percentage of lymphocytes |  |  | 1 |  |
| Increase in percentage of monocytes |  | 1 |  |  |
| Increase in blood bilirubin |  |  |  | 1 |
| Decrease in blood lactate dehydrogenase | 1 |  |  |  |
| Increase in C-reactive protein | 1 | 1 | 1 | 4 |
| Increase in creatinine phosphokinase in blood | 2 |  | 2 | 1 |
| Test positive for glucose in urine |  |  | 1 |  |
| Test positive for albumin in urine | 1 | 5 | 1 | 6 |
| Increase in β-N-acetyl-D-glucosaminidase | 2 | 3 | 1 | 1 |
| Increase in β2 microglobulin | 1 | 3 | 4 | 3 |
| Diarrhoea |  | 1 |  |  |
| Pain |  |  | 1 |  |
| Abdominal pain |  | 1* |  |  |
| Total | 10 | 19 | 14 | 17 |

*Pains experienced by 1 patient in Group M required therapy, and the patient began to experience pain at the surgical site starting 8 days after administration that resolved 35 days after administration with the use of drugs such as cefcapene pivoxil hydrochloride, lysozyme hydrochloride, rebamipide and loxoprofen sodium.
